# Supplementary material for: Experimentally broadcast ocean surf and river noise alters birdsong
Source: PeerJ. 2022 May 17;10:e13297. doi: 10.7717/peerj.13297 (PMC9121869; doi:10.7717/peerj.13297)
Supplement: Supplemental Information 1 — Mean ±SD for frequency measures are in kilohertz, duration is in seconds, and rate is in syllables or trills per second. [file peerj-10-13297-s001.docx]

|  | Song subset | | | | |  | All analyzed songs | | | | | | | | | | |
| --- | --- | --- | --- | --- | --- | --- | --- | --- | --- | --- | --- | --- | --- | --- | --- | --- | --- |
|  | Minimum frequency |  | Maximum frequency |  | Frequency bandwidth |  | Center frequency |  | 5% frequency |  | 95% frequency |  | 90% bandwidth |  | Duration |  | Syllable rate |
| Idaho | Mean  ± SD |  | Mean  ± SD |  | Mean  ± SD |  | Mean  ± SD |  | Mean  ± SD |  | Mean  ± SD |  | Mean  ± SD |  | Mean  ± SD |  | Mean  ± SD |
| Lazuli bunting | 2.94  ± 0.24 |  | 7.75  ± 0.56 |  | 4.81  ± 0.58 |  | 4.92  ± 0.32 |  | 3.61  ± 0.27 |  | 6.61  ± 0.42 |  | 3.00  ± 0.41 |  | 2.11  ± 0.52 |  | 6.21  ± 0.80 |
| Song sparrow | 2.51  ± 0.26 |  | 8.18  ± 0.67 |  | 5.67  ± 0.70 |  | 4.32  ± 0.59 |  | 2.99  ± 0.44 |  | 6.50  ± 0.87 |  | 3.50  ± 1.00 |  | 2.58  ± 0.39 |  | 5.82  ± 1.64 |
| Warbling vireo | 2.26  ± 0.20 |  | 6.05  ± 0.43 |  | 3.78  ± 0.46 |  | 3.75  ± 0.25 |  | 2.74  ± 0.17 |  | 4.96  ± 0.40 |  | 2.22  ± 0.42 |  | 1.98  ± 0.55 |  | 5.75  ± 0.91 |
| Yellow warbler | 3.61  ± 0.36 |  | 8.36  ± 0.77 |  | 4.74  ± 0.73 |  | 5.44  ± 0.41 |  | 4.22  ± 0.33 |  | 6.98  ± 0.45 |  | 2.76  ± 0.41 |  | 1.28  ± 0.21 |  | 6.83  ± 0.97 |
|  | Trill subset | | | | |  | All analyzed songs | | | | | | | | | | |
|  | Minimum frequency |  | Maximum frequency |  | Frequency bandwidth |  | Center frequency |  | 5% frequency |  | 95% frequency |  | 90% bandwidth |  | Duration |  | Trill rate |
| California | Mean  ± SD |  | Mean  ± SD |  | Mean  ± SD |  | Mean  ± SD |  | Mean  ± SD |  | Mean  ± SD |  | Mean  ± SD |  | Mean  ± SD |  | Mean  ± SD |
| White-crowned sparrow | 2.87  ± 0.17 |  | 6.69  ± 0.32 |  | 3.82  ± 0.34 |  | 4.49  ± 0.27 |  | 3.62  ± 0.27 |  | 5.50  ± 0.33 |  | 1.88  ± 0.42 |  | 1.91  ± 0.21 |  | 3.49  ± 0.28 |
| Wrentit | 2.37  ± 0.17 |  | 3.40  ± 0.16 |  | 1.03  ± 0.19 |  | 3.09  ± 0.16 |  | 2.73  ± 0.19 |  | 3.27  ± 0.15 |  | 0.54  ± 0.14 |  | 2.69  ± 0.57 |  | 11.08  ± 3.84 |
