# Supplementary material for: Experimentally broadcast ocean surf and river noise alters birdsong
Source: PeerJ. 2022 May 17;10:e13297. doi: 10.7717/peerj.13297 (PMC9121869; doi:10.7717/peerj.13297)
Supplement: Supplemental Information 2 — Phantom-off, shifted-off, and control site samples were combined into one factor level (light grey columns, differentiated for clarity). Samples of <4 individuals (dark grey cells) at positive control and shifted sites were excluded from analysis. SS (song subset) and TS (trill subset) denote the respective Idaho and California subsets with additional frequency measures collected. Centered numbers indicate no difference between the SS/TS subset and total (All) individuals. Subscripts denote number of individuals prompted to sing with conspecific playback in Idaho and number of individuals whose songs were extracted from SM3BAT recorders in California. Superscripts indicate number of individuals recorded with phantom/shifted loudspeakers both on and off. Number of songs per number of recorded individuals included for analysis is notated under species name. [file peerj-10-13297-s002.docx]

|  | Treatment type | | | | | | | | | | | | | | | | |
| --- | --- | --- | --- | --- | --- | --- | --- | --- | --- | --- | --- | --- | --- | --- | --- | --- | --- |
|  | Control | |  | Phantom-off | |  | Shifted-off | |  | Positive control | |  | Phantom | |  | Shifted | |
| Idaho | SS | All |  | SS | All |  | SS | All |  | SS | All |  | SS | All |  | SS | All |
| Lazuli bunting  (*n* = 232/52) | 11_1_ | |  | 6_3_^1^ | 7_3_^2^ |  | 7 | 9^1^ |  | 0 | 1 |  | 10^2^ | 14_2_^2^ |  | 10_3_ | 14_5_^1^ |
| Song sparrow  (*n* = 130/30) | 9_3_ | |  | 4^1^ | |  | 4 | |  | 1 | |  | 7_1_^1^ | 8_1_^1^ |  | 6_2_ | |
| Warbling vireo  (*n* = 164/34) | 10_2_ | |  | 2^1^ | |  | 2 | |  | 3_1_ | 4_1_ |  | 6_1_ | 9_1_^1^ |  | 6_1_ | 8_2_ |
| Yellow warbler  (*n* = 160/39) | 8_2_ | 9_2_ |  | 4_1_ | |  | 6 | |  | 2 | 4_1_ |  | 7_1_ | 9_2_ |  | 7_2_ | |
| California | TS | All |  | TS | All |  | TS | All |  | TS | All |  | TS | All |  | TS | All |
| White-crowned sparrow  (*n* = 281/62) | 13_6_ | 14_6_ |  | 12_1_^2^ | |  | 5_1_^1^ | |  | 16_1_ | 17_2_ |  | 8_2_^2^ | 11_3_^2^ |  | 4_1_^1^ | 6_1_^1^ |
| Wrentit  (*n* = 155/44) | 9_6_ | 12_9_ |  | 11_3_^1^ | 15_4_^2^ |  | 5_1_^1^ | 7_2_^1^ |  | 2 | 3 |  | 7_1_^1^ | 9_2_^1^ |  | 2 | 5_1_^1^ |
