# Supplementary material for: Experimentally broadcast ocean surf and river noise alters birdsong
Source: PeerJ. 2022 May 17;10:e13297. doi: 10.7717/peerj.13297 (PMC9121869; doi:10.7717/peerj.13297)
Supplement: Supplemental Information 3 — Models with Δ AICc ≤ 2.00 and the null (intercept-only) are reported. K is the number of model parameters, log(<!–[if !msEquation]–> <!–[if !vml]–> <!–[endif]–> <!–[endif]–>) is the maximized log-likelihood, AICc is the Akaike Information Criterion corrected for small sample size, Δ is the change in AIC c from the top model (0.00), wi is the Akaike weight for all strong supporting models. Parameters with 85% CIs that do not include zero are italicized and 95% CIs are in bold. Positive/negative symbols (+/ −) indicate direction of influence for variables. Greater/less than symbols (</ >) indicate direction of influence between treatment levels (C = control, PC = positive control, P = phantom, S = shifted) with an effect. Treatment is in bold, italic font if different level comparisons yield different effects (85 and 95% CIs) in the same model. Null (intercept-only) subscripts indicate the random effects structure for all models of a given song feature (ID = individual bird I.D., Site = site name, Rec = recordist and recording unit type, All = all three random intercepts, “/” indicates nesting, “+” indicates random intercepts without nesting). [file peerj-10-13297-s003.docx]

| White-crowned sparrow | *K* | log($\mathcal{L}$) | AIC*_c_* | Δ | *w_i_* |
| --- | --- | --- | --- | --- | --- |
| Minimum frequency (Trill subset): |  |  |  |  |  |
| dBA (+) | 5 | 204.93 | -399.57 | 0.00 | 0.45 |
| dBA (+), Julian date (+) | 6 | 205.74 | -399.08 | 0.49 | 0.35 |
| dBA (+), *Treatment* (*C*>*PC*, *C*>*S*) | 8 | 207.32 | -397.97 | 1.60 | 0.20 |
| Null_Site/ID_ | 4 | 202.48 | -396.77 | 2.80 | - |
| Maximum frequency (Trill subset): |  |  |  |  |  |
| Treatment (C>PC, C>S, P>PC, P>S) | 8 | 85.74 | -154.79 | 0.00 | 0.48 |
| dBA (-), *Treatment* (*C*>*PC*, C>S, P>PC, P>S) | 9 | 86.45 | -154.04 | 0.75 | 0.33 |
| Julian date (+), Treatment (C>PC, C>S, P>PC, P>S) | 9 | 85.84 | -152.83 | 1.97 | 0.18 |
| Null_All_ | 5 | 79.22 | -148.15 | 6.64 | - |
| Frequency bandwidth (Trill subset): |  |  |  |  |  |
| dBA (-) | 6 | 47.83 | -83.27 | 0.00 | 0.52 |
| dBA (-), *Treatment* (*C*<*P*, *P>PC*, *P*>*S* ) | 9 | 50.31 | -81.75 | 1.52 | 0.24 |
| dBA (-), Year (-) | 7 | 48.10 | -81.67 | 1.60 | 0.23 |
| Null_All_ | 5 | 44.99 | -79.70 | 3.57 | - |
| Center frequency: |  |  |  |  |  |
| Null_All_ | 5 | 89.66 | -169.10 | 0.00 | 0.73 |
| dBA (+) | 6 | 89.70 | -167.10 | 2.00 | 0.27 |
| 5% frequency: |  |  |  |  |  |
| Null_ID+Rec_ | 4 | 67.87 | -127.59 | 0.00 | 1.00 |
| 95% frequency: |  |  |  |  |  |
| Null_All_ | 5 | 41.48 | -72.73 | 0.00 | 0.28 |
| *Treatment* (C<S, P<S, *PC*<*S*) | 8 | 44.47 | -72.40 | 0.33 | 0.23 |
| dBA (+) | 6 | 42.05 | -71.78 | 0.95 | 0.17 |
| Year (-) | 6 | 41.58 | -70.86 | 1.88 | 0.11 |
| Julian date (-) | 6 | 41.55 | -70.79 | 1.94 | 0.10 |
| dBA (+), *Treatment* (*C*<*S*, P<S) | 9 | 44.70 | -70.74 | 1.99 | 0.10 |
| 90% frequency bandwidth: |  |  |  |  |  |
| Null_ID_ | 3 | -65.45 | 136.98 | 0.00 | 0.31 |
| Year (-) | 4 | -64.62 | 137.38 | 0.40 | 0.26 |
| Julian date (-) | 4 | -65.15 | 138.44 | 1.46 | 0.15 |
| dBA (+) | 4 | -65.18 | 138.50 | 1.52 | 0.15 |
| Julian date (-), Year (-) | 5 | -64.24 | 138.70 | 1.72 | 0.13 |
| Duration: |  |  |  |  |  |
| *Treatment* (C>PC, C>S, *P*>*S*), *Year* (+) | 8 | 195.96 | -375.39 | 0.00 | 0.36 |
| Treatment (C>PC, C>S, P>S) | 7 | 194.87 | -375.32 | 0.07 | 0.35 |
| Julian date (-), *Treatment* (C>PC, C>S, *P*>*S*), *Year* (+) | 9 | 196.09 | -373.52 | 1.86 | 0.14 |
| Julian date (-), Treatment (C>PC, C>S, P>S) | 8 | 195.00 | -373.47 | 1.91 | 0.14 |
| Null_Site/ID_ | 4 | 188.42 | -368.70 | 6.69 | - |
| Trill rate: |  |  |  |  |  |
| Null_Site/ID_ | 4 | 238.60 | -469.05 | 0.00 | 0.23 |
| dBA (+) | 5 | 239.50 | -468.79 | 0.26 | 0.20 |
| Julian date (-) | 5 | 239.34 | -468.47 | 0.58 | 0.17 |
| Year (-) | 5 | 238.91 | -467.60 | 1.44 | 0.11 |
| dBA (+), Julian date (-) | 6 | 239.87 | -467.43 | 1.62 | 0.10 |
| Julian date (-), Year (-) | 6 | 239.70 | -467.09 | 1.95 | 0.09 |
| dBA (+), Year (-) | 6 | 239.70 | -467.08 | 1.96 | 0.09 |
