# Supplementary material for: Experimentally broadcast ocean surf and river noise alters birdsong
Source: PeerJ. 2022 May 17;10:e13297. doi: 10.7717/peerj.13297 (PMC9121869; doi:10.7717/peerj.13297)
Supplement: Supplemental Information 4 — See Table S3 description for additional table details. [file peerj-10-13297-s004.docx]

| Wrentit | *K* | log($\mathcal{L}$) | AIC*_c_* | Δ | *w_i_* |
| --- | --- | --- | --- | --- | --- |
| Minimum frequency (Trill subset): |  |  |  |  |  |
| dBA (-), *Julian date* (-) | 6 | 77.74 | -142.69 | 0.00 | 0.20 |
| dBA (-), Julian date (-), *Year* (+) | 7 | 78.87 | -142.67 | 0.02 | 0.19 |
| *dBA* (-) | 5 | 76.47 | -142.37 | 0.31 | 0.17 |
| *Treatment* (*C*>*P*) | 5 | 75.99 | -141.40 | 1.28 | 0.10 |
| Null_Site/ID_ | 4 | 74.88 | -141.38 | 1.30 | 0.10 |
| Julian date (-), *Treatment* (*C*>*P*) | 6 | 76.92 | -141.03 | 1.65 | 0.09 |
| Julian date (-) | 5 | 75.72 | -140.88 | 1.81 | 0.08 |
| *dBA* (-), Year (+) | 6 | 76.80 | -140.80 | 1.88 | 0.08 |
| Maximum frequency (Trill subset): |  |  |  |  |  |
| Null_All_ | 5 | 143.48 | -276.40 | 0.00 | 0.64 |
| Year (+) | 6 | 144.02 | -275.24 | 1.16 | 0.36 |
| Frequency bandwidth (Trill subset): |  |  |  |  |  |
| Julian date (+), Treatment (C<P) | 6 | 71.37 | -129.94 | 0.00 | 0.67 |
| dBA (+), Julian date (+) | 6 | 70.65 | -128.50 | 1.44 | 0.33 |
| Null_Site/ID_ | 4 | 65.95 | -123.53 | 6.41 | - |
| Center frequency: |  |  |  |  |  |
| Null_All_ | 5 | 190.26 | -370.13 | 0.00 | 0.31 |
| Julian date (-) | 6 | 191.17 | -369.77 | 0.35 | 0.26 |
| *Julian date* (-), Year (+) | 7 | 192.13 | -369.50 | 0.63 | 0.23 |
| Year (+) | 6 | 190.88 | -369.19 | 0.93 | 0.20 |
| 5% frequency: |  |  |  |  |  |
| dBA (-), Julian date (-), Year (+) | 7 | 113.69 | -212.62 | 0.00 | 1.00 |
| Null_Site/ID_ | 4 | 99.17 | -190.08 | 22.54 | - |
| 95% frequency: |  |  |  |  |  |
| Treatment (C<S, P<S) | 6 | 212.62 | -412.67 | 0.00 | 0.38 |
| Treatment (C<S, P<S), Year (+) | 7 | 213.60 | -412.43 | 0.24 | 0.33 |
| Julian date (+), Treatment (C<S, P<S) | 7 | 212.77 | -410.79 | 1.89 | 0.15 |
| dBA (+), *Treatment* (*C*<*S*, P<S) | 7 | 212.74 | -410.71 | 1.96 | 0.14 |
| Null_Site/ID_ | 4 | 207.30 | -406.33 | 6.34 | - |
| 90% frequency bandwidth: |  |  |  |  |  |
| dBA (+), Julian date (+) | 7 | 127.04 | -239.32 | 0.00 | 0.60 |
| dBA (+), Julian date (+), Year (-) | 8 | 127.75 | -238.51 | 0.82 | 0.40 |
| Null_All_ | 5 | 119.76 | -229.12 | 10.20 | - |
| Duration: |  |  |  |  |  |
| Null_Site/ID_ | 4 | -118.52 | 245.30 | 0.00 | 0.54 |
| Year (+) | 5 | -118.20 | 246.81 | 1.51 | 0.25 |
| Julian date (+) | 5 | -118.42 | 247.24 | 1.94 | 0.20 |
| Trill rate: |  |  |  |  |  |
| Julian date (+) | 4 | -143.42 | 295.12 | 0.00 | 0.57 |
| Julian date (+), *Treatment* (*C*>*P*, *P*<*S*) | 6 | -141.58 | 295.72 | 0.60 | 0.43 |
| Null_ID_ | 3 | -148.07 | 302.30 | 7.18 | - |
