# Supplementary material for: Experimentally broadcast ocean surf and river noise alters birdsong
Source: PeerJ. 2022 May 17;10:e13297. doi: 10.7717/peerj.13297 (PMC9121869; doi:10.7717/peerj.13297)
Supplement: Supplemental Information 5 — See Table S3 description for additional table details. [file peerj-10-13297-s005.docx]

| Lazuli bunting model | *K* | log($\mathcal{L}$) | AIC*_c_* | Δ | *w_i_* |
| --- | --- | --- | --- | --- | --- |
| Minimum frequency (Song subset): |  |  |  |  |  |
| *Playback* (-) | 6 | 90.73 | -168.96 | 0.00 | 0.26 |
| Null_All_ | 5 | 89.63 | -168.91 | 0.05 | 0.26 |
| dBA (+) | 6 | 90.28 | -168.07 | 0.90 | 0.17 |
| Julian date (-), *Playback* (-) | 7 | 91.30 | -167.95 | 1.01 | 0.16 |
| dBA (+), Playback (-) | 7 | 91.27 | -167.88 | 1.09 | 0.15 |
| Maximum peak frequency contour (Song subset): |  |  |  |  |  |
| *dBA* (-) | 5 | -75.79 | 161.92 | 0.00 | 0.35 |
| Null_ID+Rec_ | 4 | -77.10 | 162.44 | 0.52 | 0.27 |
| *dBA* (-), Playback (+) | 6 | -75.20 | 162.88 | 0.96 | 0.21 |
| Playback (+) | 5 | -76.50 | 163.34 | 1.42 | 0.17 |
| Frequency bandwidth (Song subset): |  |  |  |  |  |
| dBA (-), *Playback* (+) | 5 | -85.21 | 180.78 | 0.00 | 0.49 |
| dBA (-) | 4 | -86.67 | 181.57 | 0.79 | 0.33 |
| *dBA* (-), Julian date (+), *Playback* (+) | 6 | -85.13 | 182.75 | 1.97 | 0.18 |
| Null_ID_ | 3 | -89.01 | 184.16 | 3.39 | - |
| Center frequency: |  |  |  |  |  |
| Null_All_ | 5 | 24.52 | -38.78 | 0.00 | 0.36 |
| Julian date (-) | 6 | 24.91 | -37.44 | 1.33 | 0.18 |
| *Treatment* (*C*>*P*, *P*<*S*) | 7 | 25.95 | -37.40 | 1.37 | 0.18 |
| dBA (+) | 6 | 24.62 | -36.87 | 1.90 | 0.14 |
| dBA (+), Treatment (C>P, P<S) | 8 | 26.74 | -36.84 | 1.94 | 0.14 |
| 5% frequency: |  |  |  |  |  |
| Treatment (C>P) | 5 | 78.36 | -146.46 | 0.00 | 0.30 |
| Null_ID_ | 3 | 76.26 | -146.41 | 0.05 | 0.29 |
| dBA (-) | 4 | 76.69 | -145.21 | 1.25 | 0.16 |
| Julian date (+), *Treatment* (C>P, *P*<*S*) | 6 | 78.60 | -144.82 | 1.64 | 0.13 |
| Playback (-), *Treatment* (C>P, *P*<*S*) | 6 | 78.44 | -144.51 | 1.94 | 0.11 |
| 95% frequency: |  |  |  |  |  |
| Excluded due to poor model performance | - | - | - | - | - |
| 90% frequency bandwidth: |  |  |  |  |  |
| Null_ID+Rec_ | 4 | -34.42 | 77.01 | 0.00 | 0.47 |
| dBA (+) | 5 | -33.93 | 78.12 | 1.11 | 0.27 |
| *Treatment* (*C*<*S*) | 6 | -32.88 | 78.13 | 1.12 | 0.27 |
| Duration: |  |  |  |  |  |
| dBA (-), Playback (+) | 5 | -95.69 | 201.65 | 0.00 | 0.67 |
| dBA (-), Julian date (+), Playback (+) | 6 | -95.36 | 203.10 | 1.45 | 0.33 |
| Null_ID_ | 3 | -99.41 | 204.93 | 3.28 | - |
| Syllable rate: |  |  |  |  |  |
| Julian date (-), Treatment (C>S, P>S) | 6 | -46.82 | 106.01 | 0.00 | 0.69 |
| dBA (+), *Julian date* (-), Treatment (C>S, P>S) | 7 | -46.57 | 107.65 | 1.64 | 0.31 |
| Null_ID_ | 3 | -63.32 | 132.74 | 26.73 | - |
