# Supplementary material for: Experimentally broadcast ocean surf and river noise alters birdsong
Source: PeerJ. 2022 May 17;10:e13297. doi: 10.7717/peerj.13297 (PMC9121869; doi:10.7717/peerj.13297)
Supplement: Supplemental Information 6 — See Table S3 description for additional table details. [file peerj-10-13297-s006.docx]

| Song sparrow model | *K* | log($\mathcal{L}$) | AIC*_c_* | Δ | *w_i_* |
| --- | --- | --- | --- | --- | --- |
| Minimum frequency (Song subset): |  |  |  |  |  |
| *Treatment* (C<P, *P*>*S*) | 6 | -5.17 | 23.13 | 0.00 | 0.27 |
| *Julian date* (+), Treatment (C<P, P>S) | 7 | -4.20 | 23.45 | 0.32 | 0.23 |
| Null_Site/ID_ | 4 | -7.58 | 23.52 | 0.39 | 0.22 |
| Julian date (+) | 5 | -6.89 | 24.34 | 1.21 | 0.15 |
| dBA (-), Treatment (C<P, P>S) | 7 | -4.81 | 24.67 | 1.54 | 0.13 |
| Maximum peak frequency contour (Song subset): |  |  |  |  |  |
| dBA (-) | 4 | -96.93 | 202.23 | 0.00 | 0.47 |
| dBA (-), Playback (+) | 5 | -96.14 | 202.84 | 0.61 | 0.35 |
| dBA (-), Julian date (-) | 5 | -96.81 | 204.18 | 1.95 | 0.18 |
| Null_ID_ | 3 | -100.00 | 206.21 | 3.99 | - |
| Frequency bandwidth (Song subset): |  |  |  |  |  |
| *Treatment* (C>P, *P*<*S*) | 5 | -103.34 | 217.23 | 0.00 | 0.34 |
| dBA (-), *Playback* (+) | 5 | -103.52 | 217.60 | 0.36 | 0.29 |
| dBA (-) | 4 | -104.91 | 218.18 | 0.94 | 0.21 |
| dBA (-), Treatment (C>P) | 6 | -102.96 | 218.70 | 1.47 | 0.16 |
| Null_ID_ | 3 | -107.98 | 222.18 | 4.95 | - |
| Center frequency: |  |  |  |  |  |
| Treatment (C<S, P<S) | 5 | -102.32 | 215.12 | 0.00 | 0.45 |
| dBA (+), Treatment (C<S, P<S) | 6 | -101.56 | 215.80 | 0.68 | 0.32 |
| Julian date (-), Treatment (C<S, P<S) | 6 | -101.88 | 216.44 | 1.32 | 0.23 |
| Null_ID_ | 3 | -105.63 | 217.44 | 2.32 | - |
| 5% frequency: |  |  |  |  |  |
| Null_All_ | 5 | -46.18 | 102.84 | 0.00 | 0.27 |
| Playback (-) | 6 | -45.54 | 103.76 | 0.92 | 0.17 |
| *Treatment* (*C*<*P*) | 7 | -44.50 | 103.93 | 1.09 | 0.16 |
| *dBA* (+), *Playback* (-) | 7 | -44.55 | 104.02 | 1.18 | 0.15 |
| dBA (+) | 6 | -45.80 | 104.27 | 1.43 | 0.13 |
| Playback (-), Treatment (C<P) | 8 | -43.72 | 104.63 | 1.79 | 0.11 |
| 95% frequency: |  |  |  |  |  |
| Null_ID+Rec_ | 4 | -116.06 | 240.45 | 0.00 | 0.42 |
| *Treatment* (*C*>*P*) | 6 | -114.52 | 241.73 | 1.28 | 0.22 |
| Playback (+) | 5 | -115.75 | 241.99 | 1.54 | 0.19 |
| dBA (-) | 5 | -115.87 | 242.21 | 1.77 | 0.17 |
| 90% frequency bandwidth: |  |  |  |  |  |
| *Treatment* (C>P, *P*<*S*) | 6 | -134.97 | 282.63 | 0.00 | 0.47 |
| Playback (+), Treatment (C>P) | 7 | -134.41 | 283.74 | 1.12 | 0.27 |
| dBA (+), Treatment (C>P, P<S) | 7 | -134.46 | 283.84 | 1.21 | 0.26 |
| Null_ID+Rec_ | 4 | -138.28 | 284.88 | 2.26 | - |
| Duration: |  |  |  |  |  |
| *dBA* (-) | 4 | -28.11 | 64.54 | 0.00 | 0.27 |
| *Playback* (-) | 4 | -28.25 | 64.83 | 0.29 | 0.23 |
| Null_ID_ | 3 | -29.45 | 65.09 | 0.55 | 0.21 |
| dBA (-), *Treatment* (*C*<*P*) | 6 | -26.54 | 65.76 | 1.22 | 0.15 |
| dBA (-), Playback (-) | 5 | -27.65 | 65.79 | 1.25 | 0.14 |
| Syllable rate: |  |  |  |  |  |
| Null_ID_ | 3 | -235.27 | 476.74 | 0.00 | 0.29 |
| Playback (+) | 4 | -234.29 | 476.90 | 0.16 | 0.27 |
| Julian date (+), *Playback* (+) | 5 | -233.53 | 477.55 | 0.81 | 0.20 |
| Julian date (+) | 4 | -235.07 | 478.47 | 1.73 | 0.12 |
| dBA (+) | 4 | -235.17 | 478.65 | 1.92 | 0.11 |
