# Supplementary material for: Experimentally broadcast ocean surf and river noise alters birdsong
Source: PeerJ. 2022 May 17;10:e13297. doi: 10.7717/peerj.13297 (PMC9121869; doi:10.7717/peerj.13297)
Supplement: Supplemental Information 7 — See Table S3 description for additional table details. [file peerj-10-13297-s007.docx]

| Warbling vireo | *K* | log($\mathcal{L}$) | AIC*_c_* | Δ | *w_i_* |
| --- | --- | --- | --- | --- | --- |
| Minimum frequency (Song subset): |  |  |  |  |  |
| Julian date (-), Treatment (C>S, P>S) | 7 | 50.28 | -85.47 | 0.00 | 0.32 |
| dBA (+), *Julian date* (-), Treatment (C>S, P>S) | 8 | 51.14 | -84.88 | 0.60 | 0.23 |
| dBA (+), Treatment (C>S, P>S) | 7 | 49.89 | -84.69 | 0.78 | 0.21 |
| *Julian date* (-), Playback (+), Treatment (C>S, P>S) | 8 | 50.47 | -83.52 | 1.95 | 0.12 |
| Treatment (C>S, P>S) | 6 | 48.15 | -83.49 | 1.98 | 0.12 |
| Null_Site/ID_ | 4 | 41.56 | -74.75 | 10.72 | - |
| Maximum peak frequency contour (Song subset): |  |  |  |  |  |
| *Julian date* (-) | 5 | -50.10 | 110.77 | 0.00 | 0.21 |
| Null_ID+Rec_ | 4 | -51.39 | 111.15 | 0.38 | 0.18 |
| *Playback* (+) | 5 | -50.34 | 111.24 | 0.47 | 0.17 |
| *dBA* (+) | 5 | -50.39 | 111.35 | 0.58 | 0.16 |
| dBA (+), Julian date (-) | 6 | -49.75 | 112.30 | 1.53 | 0.10 |
| Julian date (-), Playback (+) | 6 | -49.77 | 112.36 | 1.59 | 0.10 |
| dBA (+), Playback (+) | 6 | -49.93 | 112.67 | 1.90 | 0.08 |
| Frequency bandwidth (Song subset): |  |  |  |  |  |
| Null_ID+Rec_ | 4 | -60.18 | 128.74 | 0.00 | 0.44 |
| dBA (+) | 5 | -59.90 | 130.38 | 1.64 | 0.19 |
| Julian date (-) | 5 | -59.92 | 130.42 | 1.68 | 0.19 |
| Playback (+) | 5 | -59.95 | 130.46 | 1.73 | 0.18 |
| Center frequency: |  |  |  |  |  |
| Julian date (-) | 6 | 6.17 | 0.20 | 0.00 | 0.39 |
| Julian date (-), *Treatment* (C<PC, P<PC, *PC*>*S*) | 9 | 9.19 | 0.80 | 0.60 | 0.29 |
| dBA (-), Julian date (-) | 7 | 6.44 | 1.84 | 1.64 | 0.17 |
| Julian date (-), Playback (-) | 7 | 6.30 | 2.12 | 1.92 | 0.15 |
| Null_All_ | 5 | 2.83 | 4.73 | 4.53 | - |
| 5% frequency: |  |  |  |  |  |
| *Playback* (+), *Treatment* (C>P, C>S, *PC*>*S*) | 8 | 85.56 | -154.19 | 0.00 | 0.28 |
| *Treatment* (C>P, C>S, *P*<*PC*, *PC*>*S*) | 7 | 84.40 | -154.08 | 0.11 | 0.27 |
| *Playback* (+) | 5 | 81.92 | -153.46 | 0.73 | 0.20 |
| Null_Site/ID_ | 4 | 80.52 | -152.79 | 1.40 | 0.14 |
| Julian date (-), *Treatment* (C>P, C>S, *P*<*PC*, *PC*>*S*) | 8 | 84.66 | -152.38 | 1.81 | 0.11 |
| 95% frequency: |  |  |  |  |  |
| Julian date (-) | 5 | -55.33 | 121.03 | 0.00 | 0.71 |
| dBA (+), Julian date (-) | 6 | -55.12 | 122.78 | 1.75 | 0.29 |
| Null_ID+Rec_ | 4 | -58.11 | 124.48 | 3.45 | - |
| 90% frequency bandwidth: |  |  |  |  |  |
| *Julian date* (-) | 5 | -56.31 | 123.01 | 0.00 | 0.43 |
| Null_ID+Rec_ | 4 | -57.97 | 124.20 | 1.19 | 0.24 |
| dBA (+), *Julian date* (-) | 6 | -56.11 | 124.75 | 1.74 | 0.18 |
| *Julian date* (-), Playback (-) | 6 | -56.21 | 124.96 | 1.95 | 0.16 |
| Duration: |  |  |  |  |  |
| Null_ID_ | 3 | -85.38 | 176.91 | 0.00 | 0.17 |
| *Julian date* (+), *Treatment* (C>P, *P*<*PC*, P<S) | 7 | -81.19 | 177.09 | 0.18 | 0.16 |
| Julian date (+) | 4 | -84.50 | 177.25 | 0.35 | 0.15 |
| *Julian date* (+), Playback (+) | 5 | -83.57 | 177.52 | 0.62 | 0.13 |
| *Treatment* (*C>P*, *P*<*PC*, P<S) | 6 | -82.53 | 177.59 | 0.68 | 0.12 |
| Julian date (+), Playback (+), *Treatment* (C>P, *P*<*PC*, P<S) | 8 | -80.37 | 177.67 | 0.76 | 0.12 |
| Playback (+) | 4 | -85.19 | 178.64 | 1.73 | 0.07 |
| *Julian date* (+), dBA (+), *Treatment* (C>P, *P*<*PC*, P<S) | 8 | -80.87 | 178.67 | 1.77 | 0.07 |
| Syllable rate: |  |  |  |  |  |
| *Julian date* (-) | 4 | -128.07 | 264.39 | 0.00 | 0.29 |
| Null_ID_ | 3 | -129.18 | 264.50 | 0.11 | 0.28 |
| *Treatment* (*C*>*S*, P>S) | 6 | -126.58 | 265.69 | 1.30 | 0.15 |
| *Julian date* (-), *Treatment* (*C*>*S*, P>S) | 7 | -125.54 | 265.80 | 1.41 | 0.15 |
| *Julian date* (-), Playback (-) | 5 | -127.84 | 266.07 | 1.68 | 0.13 |
