# Supplementary material for: Experimentally broadcast ocean surf and river noise alters birdsong
Source: PeerJ. 2022 May 17;10:e13297. doi: 10.7717/peerj.13297 (PMC9121869; doi:10.7717/peerj.13297)
Supplement: Supplemental Information 8 — See Table S3 description for additional table details. ATreatment had a negative influence relative to control reference condition; no level comparisons yielded an effect. [file peerj-10-13297-s008.docx]

| Yellow warbler | *K* | log($\mathcal{L}$) | AIC*_c_* | Δ | *w_i_* |
| --- | --- | --- | --- | --- | --- |
| Minimum frequency (Song subset): |  |  |  |  |  |
| *Julian date* (-) | 6 | -16.20 | 45.14 | 0.00 | 0.40 |
| Null_All_ | 5 | -17.87 | 46.27 | 1.13 | 0.23 |
| Julian date (-), *Treatment* (*P*<*S*) | 8 | -14.57 | 46.45 | 1.31 | 0.21 |
| Julian date (-), Playback (-) | 7 | -15.96 | 46.94 | 1.80 | 0.16 |
| Maximum peak frequency contour (Song subset): |  |  |  |  |  |
| Null_Site/ID_ | 4 | -97.36 | 203.08 | 0.00 | 0.53 |
| Julian date (-) | 5 | -97.00 | 204.53 | 1.46 | 0.26 |
| Playback (+) | 5 | -97.16 | 204.86 | 1.78 | 0.22 |
| Frequency bandwidth (Song subset): |  |  |  |  |  |
| Null_ID+Rec_ | 4 | -95.65 | 199.66 | 0.00 | 0.45 |
| Playback (+) | 5 | -95.38 | 201.29 | 1.63 | 0.20 |
| Treatment (-)^a^ | 6 | -94.36 | 201.46 | 1.81 | 0.18 |
| Julian date (-) | 5 | -95.51 | 201.55 | 1.89 | 0.17 |
| Center frequency: |  |  |  |  |  |
| Julian date (-), Playback (-) | 5 | -35.15 | 80.69 | 0.00 | 0.71 |
| Julian date (-) | 4 | -37.12 | 82.50 | 1.80 | 0.29 |
| Null_ID_ | 3 | -45.17 | 96.49 | 15.79 | - |
| 5% frequency: |  |  |  |  |  |
| *dBA* (-), Julian date (-) | 5 | -12.10 | 34.60 | 0.00 | 0.33 |
| Julian date (-) | 4 | -13.30 | 34.86 | 0.26 | 0.29 |
| Julian date (-), Playback (-) | 5 | -12.68 | 35.76 | 1.16 | 0.19 |
| Julian date (-), *dBA* (-), Playback (-) | 6 | -11.61 | 35.77 | 1.18 | 0.19 |
| Null_ID_ | 3 | -18.75 | 43.65 | 9.05 | - |
| 95% frequency: |  |  |  |  |  |
| Julian date (-), *Playback* (-) | 5 | -51.17 | 112.72 | 0.00 | 0.48 |
| Julian date (-) | 4 | -52.56 | 113.38 | 0.66 | 0.34 |
| *dBA* (+), Julian date (-), Playback (-), *Treatment* (C>P, *C*>*PC*, P<S, *PC*<*S*) | 9 | -47.71 | 114.62 | 1.90 | 0.18 |
| Null_ID_ | 3 | -56.57 | 119.30 | 6.58 | - |
| 90% frequency bandwidth: |  |  |  |  |  |
| Null_ID_ | 3 | -64.64 | 135.44 | 0.00 | 0.30 |
| dBA (+) | 4 | -63.90 | 136.06 | 0.61 | 0.22 |
| Julian date (-) | 4 | -64.43 | 137.12 | 1.68 | 0.13 |
| Playback (-) | 4 | -64.49 | 137.24 | 1.80 | 0.12 |
| dBA (+), Treatment (C>P) | 7 | -61.27 | 137.29 | 1.84 | 0.12 |
| dBA (+), Playback (-) | 5 | -63.52 | 137.44 | 2.00 | 0.11 |
| Duration: |  |  |  |  |  |
| Julian date (+), Playback (+), *Treatment* (C<P, *P*>*PC*, P>S) | 10 | 93.02 | -164.57 | 0.00 | 0.44 |
| Julian date (+), Playback (+) | 7 | 89.48 | -164.23 | 0.34 | 0.37 |
| Playback (+) | 6 | 87.74 | -162.93 | 1.65 | 0.19 |
| Null_All_ | 5 | 84.51 | -158.62 | 5.95 | - |
| Syllable rate: |  |  |  |  |  |
| Julian date (-) | 5 | -139.52 | 289.44 | 0.00 | 0.55 |
| Julian date (-), Playback (-) | 6 | -139.25 | 291.06 | 1.62 | 0.24 |
| dBA (+), Julian date (-) | 6 | -139.43 | 291.40 | 1.96 | 0.21 |
| Null_Site/ID_ | 4 | -143.60 | 295.46 | 6.02 | - |
