# Supplementary material for: Experimentally broadcast ocean surf and river noise alters birdsong
Source: PeerJ. 2022 May 17;10:e13297. doi: 10.7717/peerj.13297 (PMC9121869; doi:10.7717/peerj.13297)
Supplement: Supplemental Information 9 — Effect sizes (β) ±SE and 95% CIs are shaded red for California comparisons and blue for Idaho comparisons. Reference conditions are listed in the top row of corresponding columns for California and the left column of corresponding rows for Idaho. Sound level (dBA) differed strongly between all treatment types at each study area except phantom and shifted in California, and positive control and phantom in Idaho (black bordered cells). [file peerj-10-13297-s009.docx]

| Acoustic condition | Statistic | Control | Positive control | Phantom | Shifted |
| --- | --- | --- | --- | --- | --- |
| Control | β ± SE |  | 11.14 ± 2.72 | 9.33 ± 1.28 | 3.52 ± 1.49 |
|  | 95% CI |  | 5.80 – 16.48 | 6.82 – 11.84 | 0.60 – 6.45 |
| Positive control | β ± SE | 3.96 ± 1.41 |  | -1.81 ± 2.84 | -7.62 ± 2.90 |
|  | 95% CI | 1.21 – 6.72 |  | -7.34 – 3.75 | -13.31 – -1.93 |
| Phantom | β ± SE | 11.11 ± 1.31 | 7.14 ± 1.70 |  | -5.81 ± 1.73 |
|  | 95% CI | 8.54 – 13.67 | 3.81 – 10.48 |  | -9.20 – -2.42 |
| Shifted | β ± SE | 12.41 ± 1.67 | 8.45 ± 1.99 | 1.31 ± 1.93 |  |
|  | 95% CI | 9.14 – 15.69 | 4.54 – 12.36 | -2.48 – 5.09 |  |
